# Supplementary material for: The FIGO ovulatory disorders classification system
Source: Int J Gynaecol Obstet. 2022 Aug 19;159(1):1–20. doi: 10.1002/ijgo.14331 (PMC10086853; doi:10.1002/ijgo.14331)
Supplement: Supplementary file 2 — Appendix S1: Supporting information [file IJGO-159-1-s002.zip › Ovulatory Disorders for Patients_English.pdf]

Ovulatory Disorders for Patients
Patient Version v.2.2 April 2022

What is ovulation?
Ovulation is when the ovary releases a tiny egg, generally midway between the starting day of one menstrual period and the start of the next period. This egg may be picked up by the fallopian tube and fertilized by a sperm, resulting in a pregnancy. If a pregnancy does not occur, the egg simply dissolves.

How does ovulation work?
The process is summarized in Figure 1. It starts with two joined organs in the brain: the hypothalamus (1) and the pituitary gland (2). The hypothalamus sends hormonal messages to the pituitary gland that, in turn, sends hormonal stimulation to a follicle (3) on one of the ovaries (4). The follicle is the structure that contains the tiny egg (5) and is also responsible for producing estrogen and, after ovulation, also progesterone. These hormones stimulate the endometrium (6) (the lining of the uterus (womb) (7) to allow a pregnancy to implant. If a pregnancy does not occur, the ovary stops producing estrogen and progesterone, and due to the fall in hormone levels, a menstrual period (menstruation) occurs. During menstruation, the upper layer of the endometrium falls off with accompanying blood and other fluid.

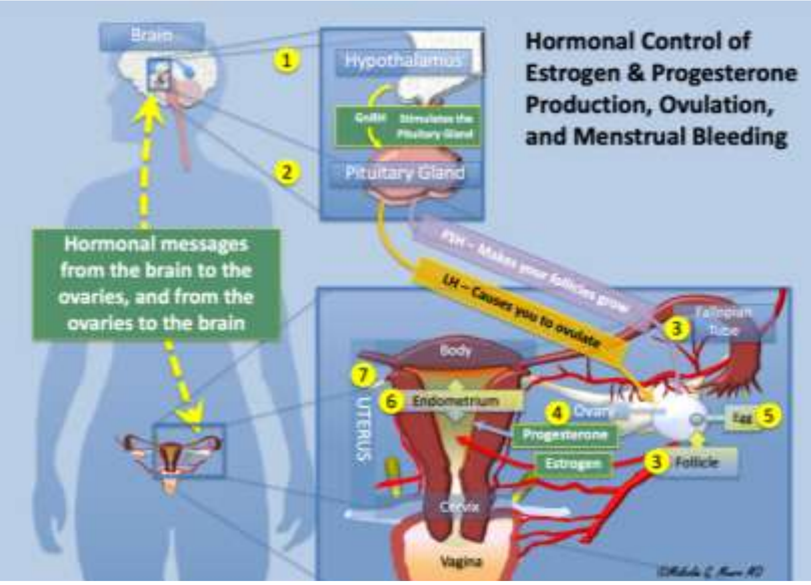

Figure 1. The structures involved in ovulation and menstruation.

What then is an ovulatory disorder?
An ovulatory disorder occurs when ovulation doesn't happen normally. Of course, ovulation doesn't normally occur if one is pregnant, breastfeeding, or using hormonal birth control such as pills or patches. There are many types of ovulatory disorders, but one way to think of them is occasional episodes or an enduring process that can last for a long time – sometimes for months or years – until a diagnosis of the cause is made and appropriate treatment is started.

What problems do ovulatory disorders cause?
There are two basic types of problems. The first is a change in the menstrual cycle. Some women and adolescent girls have no periods, a condition called "amenorrhea," while others have infrequent or irregular menstrual bleeding (irregular timing of periods). The second, of course, is difficulty getting pregnant, a condition called "subfertility" or "infertility."

What causes ovulatory disorders?
There are many causes of ovulatory disorders, some of which are temporary and return to normal, others that require treatments to correct the problem. For example, shortly after the start of a period and shortly before menopause, ovulatory disorders are very common. During the teen years, usually, the condition sorts itself out without the need for treatment. In older women, typically in their late 40s or early 50s, the "sorting out" comes at menopause, when periods stop as the ovaries stop making estrogen. Because there are so many possible causes of ovulatory disorders, the International Federation of Gynecology and Obstetrics (FIGO) has developed a system to aid health care providers, teachers, and researchers improve the quality of health care and the education of everyone involved. It is known as HyPO-P, and we will explain how it got that name below.

Ovulatory Disorders for Patients

How does this FIGO Ovulatory Disorders System work?

Health care providers use important information obtained from the patient to help determine whether there is an ovulatory disorder. This history is combined with a physical examination, appropriate lab tests, and often, imaging of the ovaries and the brain to determine the potential cause. The new Ovulatory Disorders System is designed to help health care providers, trainees, and researchers speak amongst each other in a common language in a way that helps standardize treatment and supports researchers. Yes, researchers. There is much about ovulatory disorders that is unknown.

The system (Figure 2) includes four main categories. Three of the four categories reflect the anatomy that has been described above: Type 1 is the hypothalamus; Type II reflects problems in the pituitary gland; and Type III problems in the ovary. The fourth, Type IV, is Polycystic Ovary Syndrome (PCOS). These causes can be remembered by the acronym HyPO-P. For each of Types I, II, and III, there are several subcategories of possible causes.

You might think that PCOS is caused by a problem in the ovary, but that is not the case – the ovary is responding to abnormal stimulation from elsewhere in the body. The ovary responds to abnormal hormonal stimulation in a way that often alters ovulation and increases the amount of male hormone circulating in the body. Many other issues can be associated with PCOS, including increased weight, acne, and even an increased risk of diabetes. Regardless, health care providers will generally place patients in one of the four categories after individual evaluation, as described above.

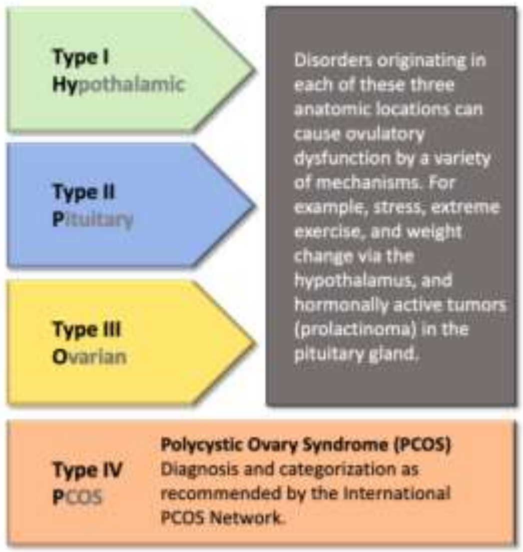

Figure 2. The FIGO Ovulatory Disorders Classification System - HyPO-P. The first three categories are based on organs in the body - disorders in any of these organs can cause ovulatory disorders. PCOS is a separate category, not considered an ovarian cause.

What are the treatments for ovulatory disorders?

In general, treatment is related to an individual patient's condition. For example, suppose the patient is trying to become pregnant and is infertile because of an ovulatory disorder. In that case, there will be a discussion about how to cause ovulation to occur, generally using medications. On the other hand, if not trying to become pregnant, those sorts of therapy may not be appropriate. Suppose irregular bleeding is a problem, or symptoms often associated with PCOS like acne or hair growth are experienced. In that case, the health care provider may suggest appropriate medications designed to help those problems. Occasionally there is a specific cause, such as an abnormal thyroid, medication taken for other reasons (each Type I cause), or the existence of a small tumor in the pituitary called a prolactinoma (a Type II cause). For these, specific approaches are recommended – treating the thyroid disorder, changing the medication, or taking a particular medication that can treat the small tumor.

Here are some important links to understand more about ovulatory disorders, including PCOS

- Resolve: The national infertility association
- Medline Plus: A website maintained by the US federal government
- Merck Manual on abnormal uterine bleeding associated with ovulatory disorders (AUB-O)
