# Supplementary material for: The FIGO ovulatory disorders classification system
Source: Int J Gynaecol Obstet. 2022 Aug 19;159(1):1–20. doi: 10.1002/ijgo.14331 (PMC10086853; doi:10.1002/ijgo.14331)
Supplement: Supplementary file 2 — Appendix S1: Supporting information [file IJGO-159-1-s002.zip › Trastornos ovulatorios para pacientes_Spanish.pdf]

Trastornos ovulatorios para pacientes
Versión para pacientes v.2.2 abril 2022

¿Qué es la ovulación?

La ovulación es el proceso en el cual el ovario libera un óvulo y, generalmente, sucede en la mitad del ciclo menstrual; entre el día de inicio de un período menstrual y el comienzo del siguiente período. Este óvulo puede ser recogido por la trompa de Falopio y fertilizado por un espermatozoide que resulta en un embarazo. Si no se produce un embarazo, el óvulo simplemente se disuelve.

¿Cómo funciona la ovulación?

Se resume el proceso en Figura 1. Comienza con dos órganos ubicados en el cerebro, el hipotálamo (1) y la glándula pituitaria (2). El hipotálamo envía mensajes hormonales a la glándula pituitaria que, a su vez, envía estimulación hormonal para inducir el crecimiento de los folículos (3) en uno de los dos ovarios (4). El folículo es el estructura que contiene el óvulo (5) también es responsable de producir estrógeno y, después de la ovulación, además progesterona. Estas hormonas estimulan el endometrio (6) (el revestimiento del útero (matriz)) (7) para permitir que se dé el proceso de implantación y el embarazo como tal. Si no se produce un embarazo, el ovario deja de producir estrógeno y progesterona, y debido a la caída de los niveles hormonales, ocurre la menstruación. Durante la menstruación, la capa superior del endometrio se cae con la sangre y otros líquidos que la acompañan.

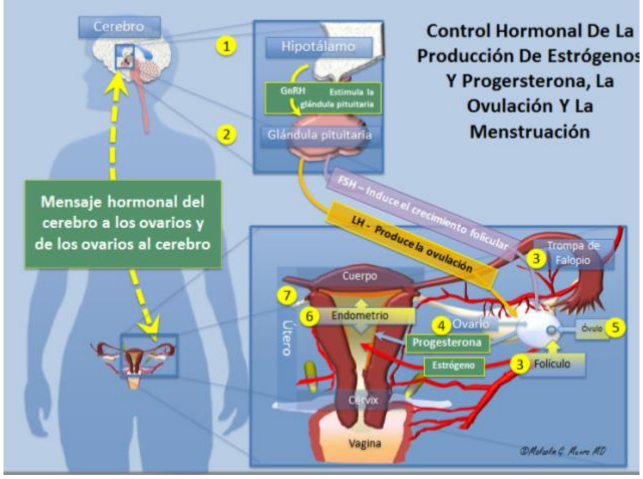

Figura 1. Las estructuras involucradas en la ovulación y la menstruación

¿Qué es entonces un trastorno ovulatorio?

El proceso de ovulación ocurre mensualmente en la edad reproductiva de la mujer, exceptuando en estado de embarazo, el periodo de lactancia, o durante el uso de algunos métodos de planificación como las píldoras o parches anticonceptivos. Sin embargo, hay mujeres que cursan con trastornos de la ovulación, en donde esta no sucede de normalmente o no ocurre en lo absoluto. Esto puede suceder de manera ocasional o ser parte de un proceso de larga duración, a veces durante meses o años, hasta ser tratado adecuadamente.

¿Qué problemas causan los trastornos ovulatorios?

Los problemas que se presentan a causa de los trastornos ovulatorios, se pueden incluir en 2 alteraciones básicas; La primera son las irregularidades en el ciclo menstrual. Algunas mujeres pueden cursar con una condición llamada amenorrea, que es la ausencia de la menstruación. Y otras pueden presentar menstruaciones poco frecuentes o irregulares. La segunda alteración es la dificultad para lograr un embarazo, esta condición se llama "infertilidad" o "subfertilidad".

¿Qué causa trastornos ovulatorios?

Hay muchas causas de trastornos ovulatorios, algunas de las cuales son temporales, y otras de larga data que requieren tratamientos específicos para corregir el problema. Por ejemplo, poco después del inicio de un período y poco antes de la menopausia, los trastornos ovulatorios son muy comunes. Durante la adolescencia, por lo general, la afección se resuelve por sí sola sin necesidad de tratamiento. En las mujeres mayores, generalmente a finales de los 40 o principios de los 50, los trastornos de la ovulación hacen parte de la transición a la menopausia, cuando los períodos menstruales se detienen a medida que los ovarios dejan de producir estrógenos. Debido a que hay tantas causas posibles de trastornos ovulatorios, la Federación Internacional de Ginecología y Obstetricia (FIGO) ha desarrollado un sistema para ayudar a los proveedores de atención médica, maestros e investigadores a mejorar la

calidad de la atención médica y la educación de todos los involucrados. Se conoce como HyPO-P, y explicaremos cómo obtuvo ese nombre a continuación.

¿Cómo funciona este sistema de trastornos ovulatorios FIGO?

Los proveedores de atención en salud utilizan información importante obtenida de la paciente para ayudar a determinar si hay un trastorno ovulatorio. Esta historia se combina con un examen físico, pruebas de laboratorio apropiadas y, a menudo, imágenes de los ovarios y el cerebro para determinar la causa potencial. El nuevo Sistema de Trastornos Ovulatorios está diseñado para ayudar a los proveedores de atención médica, aprendices e investigadores a hablar entre sí en un idioma común de una manera que ayude a estandarizar el tratamiento y que apoye a los investigadores. Sí, investigadores. Hay mucho sobre los trastornos ovulatorios que se desconoce.

El sistema (Figura 2) incluye cuatro categorías principales. Tres de las cuatro categorías reflejan la anatomía que se ha descrito anteriormente: en el tipo I se incluyen las causas de origen hipotalámico; el tipo II refleja problemas en la glándula pituitaria y problemas de tipo III en el ovario. El tipo IV es el SOP (síndrome de ovario poliquístico). Estas causas pueden ser recordadas por el acrónimo HyPO-P. Para cada uno de los tipos I, II y III, hay varias subcategorías de posibles causas.

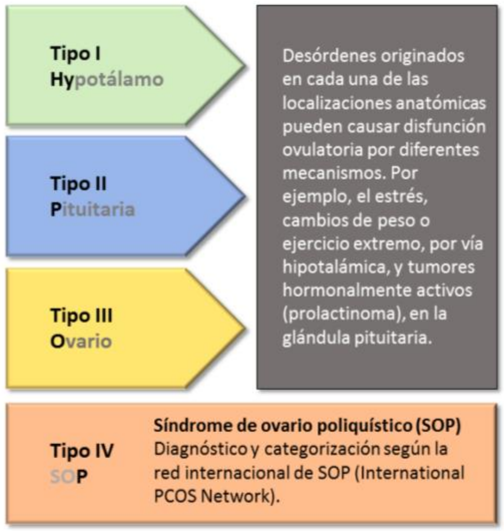

categorías se basan en los órganos del cuerpo: los trastornos en cualquiera de estos órganos pueden causar trastornos ovulatorios. El SOP es una categoría separada, no se considera una causa ovárica

Podrías pensar que SOP es causada por un problema en el ovario, y por tanto corresponder al tipo III, pero ese no es el caso – el ovario está respondiendo a una estimulación anormal proveniente de otra parte en el cuerpo. El ovario responde a la estimulación hormonal anormal de una manera que a menudo altera la ovulación y aumenta la cantidad de hormona masculina que circula en el cuerpo (andrógenos). Existen otros problemas asociados al SOP, más allá del trastorno ovulatorio, incluido aumento de peso, acné, e incluso un mayor riesgo de diabetes. En cualquier caso, la atención médica proporcionada generalmente clasificará a las pacientes en una de las cuatro categorías después de evaluación individual, como se describió anteriormente.

¿Cuáles son los tratamientos para los trastornos ovulatorios?

En general, el tratamiento está relacionado con la condición de un paciente individual. Por ejemplo, supongamos que la paciente está tratando de quedar embarazada y es infértil debido a un trastorno ovulatorio. En ese caso, habrá una discusión sobre cómo inducir la ovulación, generalmente usando medicamentos. Por otro lado, si no está tratando de quedar embarazada, ese tipo de terapia puede no ser apropiada. Supongamos que el problema es el sangrado menstrual irregular o los síntomas a menudo asociados con el SOP, como el acné o el crecimiento del vello en áreas no deseadas. En ese caso, el proveedor de atención médica puede sugerir medicamentos apropiados diseñados para ayudar a esos problemas. Ocasionalmente hay una causa específica, como un funcionamiento anormal de la glándula tiroides, medicamentos tomados por otras razones (cada causa tipo I) o la existencia de un pequeño tumor en la hipófisis llamado prolactinoma (una causa tipo II). Para estos, se recomiendan enfoques específicos: tratar el trastorno de la tiroides, cambiar el medicamento o tomar un medicamento en particular que pueda tratar el tumor pequeño.

Aquí hay algunos enlaces importantes para comprender más sobre los trastornos ovulatorios, incluido el SOP

- Resolver: La asociación nacional de infertilidad
- Medline Plus: Un sitio web mantenido por el gobierno federal
- Manual de Merk sobre sangrado uterino anormal asociado con trastornos ovulatorios (AUB-O)
