# Supplementary material for: The FIGO ovulatory disorders classification system
Source: Int J Gynaecol Obstet. 2022 Aug 19;159(1):1–20. doi: 10.1002/ijgo.14331 (PMC10086853; doi:10.1002/ijgo.14331)
Supplement: Supplementary file 2 — Appendix S1: Supporting information [file IJGO-159-1-s002.zip › Troubles ovulatoires pour les patients_French.pdf]

Troubles ovulatoires pour les patients

Version patient v. 2.2 avril 2022

Qu'est-ce que l'ovulation?

L'ovulation se produit lorsque l'ovaire libère un minuscule ovule, généralement à mi-chemin entre le début d'un cycle menstruel et le début du cycle suivant. Cet ovule peut être capté par la trompe de Fallope et fécondé par un spermatozoïde entraînant une grossesse. Si une grossesse ne se produit pas, l'ovule se dissout simplement.

Comment fonctionne l'ovulation ?

Le processus est résumé dans la Figure 1. Cela débute par deux organes proches du cerveau, l'hypothalamus (1) et l'hypophyse (2). L'hypothalamus envoie des messages hormonaux à l'hypophyse qui envoie à son tour une stimulation hormonale à un follicule (3) sur l'un des ovaires (4). Le follicule est une structure qui contient le minuscule œuf, ou ovule, (5) et est responsable de la production d'œstrogènes mais également, après l'ovulation, de progestérone. Ces hormones stimulent l'endomètre (6), qui est la muqueuse ou couche interne de l'utérus (7), pour permettre à une grossesse de s'implanter. Si une grossesse ne se produit pas, l'ovaire cesse de produire œstrogènes et progestérone, et en raison de la baisse des niveaux hormonaux, un nouveau cycle menstruel débute et les menstruations (règles) surviennent. Lors des menstruations, la couche superficielle de l'endomètre desquame, accompagnée de sang et d'autres liquides.

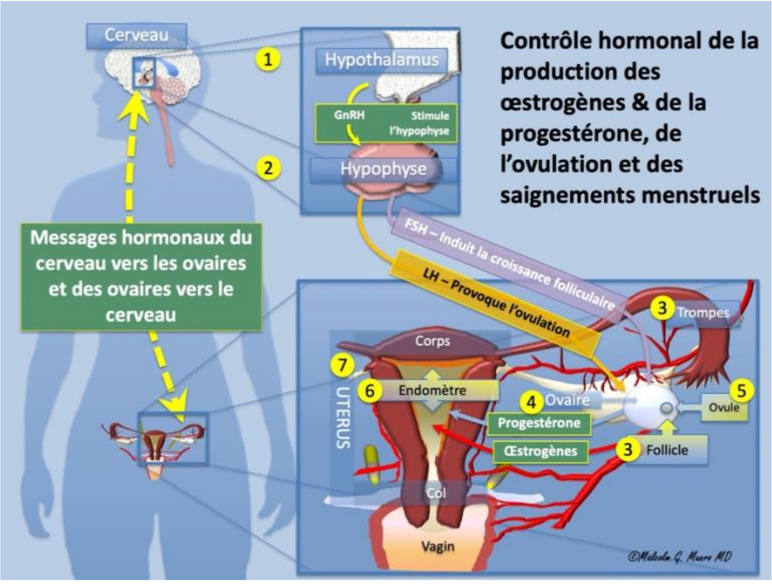

Graphique 1. Les structures impliquées dans l'ovulation et les menstruations

Qu'est-ce donc qu'un trouble ovulatoire ?

Un trouble ovulatoire se produit lorsque l'ovulation ne se produit pas normalement. Bien sûr, de façon normale, l'ovulation ne se produit pas si on est enceinte, qu'on allaite ou qu'on utilise une contraception hormonale tels que les pilules ou les patchs. Il existe de nombreux types de troubles ovulatoires, mais une façon de les envisager est de différentier les épisodes occasionnels d'un processus durable qui peut durer longtemps – parfois pendant des mois ou des années, jusqu'à ce qu'un diagnostic de la cause soit posé et qu'un traitement approprié soit débuté.

Quels problèmes les troubles ovulatoires causent-ils ?

Il existe deux types de problèmes. Le premier est un changement dans le cycle menstruel. Certaines femmes et adolescentes ne sont pas réglées, c'est une « aménorrhée », tandis que d'autres ont des saignements menstruels peu fréquents ou irréguliers. La seconde, bien sûr, est la difficulté à obtenir une grossesse, c'est une « hypofertilité » ou une « infertilité ».

Qu'est-ce qui provoque des troubles ovulatoires ?

Il existe de nombreuses causes de troubles ovulatoires, dont certaines sont temporaires et permettent un retour à la normale alors que d'autres peuvent nécessiter des mesures thérapeutiques afin de corriger le problème. Par exemple, peu de temps après l'apparition des premières règles et avant la ménopause, les troubles ovulatoires sont très fréquents. Pendant l'adolescence, généralement, cette irrégularité menstruelle se règle d'elle-même sans nécessiter de traitement. Chez les femmes plus âgées, généralement à la fin de la quarantaine ou au début de la cinquantaine, la « résolution » vient à la ménopause, lorsque les règles s'arrêtent quand les ovaires cessent de produire des œstrogènes. C'est parce qu'il y a énormément de causes possibles de troubles ovulatoires que la

Fédération Internationale de Gynécologie et d'Obstétrique (FIGO) a développé un système pour aider les prestataires de soins de santé, les enseignants et les chercheurs à améliorer la qualité des soins de santé prodigués aux patientes et l'éducation de toutes les personnes impliquées. Il est connu sous le nom de HyPO-P, et nous expliquerons comment ce nom a été obtenu ci-dessous.

Comment fonctionne ce système de classification des troubles ovulatoires FIGO ?
Les prestataires de soins de santé utilisent des informations importantes obtenues de la part du patient pour aider à déterminer s'il existe ou non un trouble ovulatoire. Ces antécédents sont combinés à un examen physique, à des tests de laboratoire appropriés et, souvent, à l'imagerie des ovaires et du cerveau pour déterminer la cause potentielle. Le nouveau système de classification des troubles ovulatoires est conçu pour aider les prestataires de soins de santé, les stagiaires et les chercheurs à parler entre eux dans un langage commun de façon à standardiser les traitements mais également à soutenir les chercheurs. Les chercheurs ? Oui ! Il y a beaucoup de choses sur les troubles ovulatoires qui restent inconnues.

Le système (figure 2) comprend quatre catégories principales. Trois des quatre catégories reflètent l'anatomie décrite ci-dessus: le type 1 est l'hypothalamus ; le type II reflète des problèmes dans l'hypophyse (*pituitary* en anglais) et le type III concerne les problèmes ovariens. Le type IV est le syndrome des ovaires polykystiques, SOPK ou OMPK en anglais. Ces causes peuvent être mémorisées par l'acronyme HyPO-P. Pour les types I, II et III, il existe plusieurs sous-catégories de causes possibles.

Vous pourriez penser que SOPK est causée par un problème dans l'ovaire, mais ce n'est pas le cas – l'ovaire répond à une stimulation anormale provenant d'une autre partie du corps. Cette réponse ovarienne est à l'origine de troubles ovulatoires et d'une augmentation de la quantité d'hormones mâles circulantes dans le corps. Beaucoup d'autres problèmes peuvent être associés au SOPK, y compris une augmentation du poids, l'apparition d'acné, et même un risque accru de diabète. Quoi qu'il en soit, les prestataires de soins de santé placeront la patiente dans l'une des quatre catégories après évaluation individuelle, comme décrit ci-dessus.

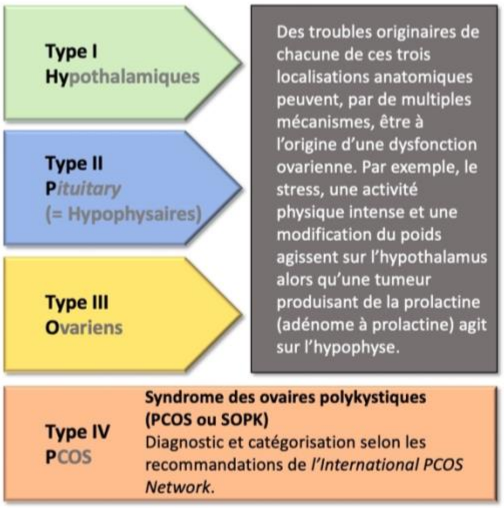

Figure 2. Le système de classification des troubles ovulatoires FIGO - HyPO-P. Les trois premières catégories sont basées sur les organes du corps - les troubles de l'un de ces organes peuvent provoquer des troubles ovulatoires. Le SOPK est une catégorie distincte, non considérée comme une cause ovarienne

Quels sont les traitements pour les troubles ovulatoires ?
En général, le traitement est lié à l'état individuel de la patiente. Par exemple, supposons que la patiente essaie de tomber enceinte et qu'elle soit infertile à cause d'un trouble ovulatoire. Dans ce cas, il y aura une discussion sur la façon d'induire l'ovulation, généralement en utilisant des médicaments. D'un autre côté, s'il n'y a pas de désir de grossesse, ce type de traitement n'est pas adéquat. Supposons que le problème principal de la patiente soit des saignements irréguliers ou des symptômes associés au SOPK comme l'acné ou un problème de pilosité. Dans ce cas, le prestataire de soins de santé peut suggérer des médicaments appropriés pour ces problèmes particuliers. Parfois, il existe une cause spécifique, tels qu'une thyroïde anormale, des médicaments pris pour d'autres raisons (chaque cause de type I) ou encore l'existence d'une petite tumeur dans l'hypophyse appelée prolactinome (une cause de type II). Pour ceux-ci, des approches spécifiques sont recommandées – traiter le trouble thyroïdien, changer le médicament ou prendre un médicament spécifique pouvant traiter la petite tumeur.

Voici quelques liens importants pour en savoir plus sur les troubles ovulatoires, y compris le SOPK

- Résolution : L'association nationale d'infertilité
- Medline Plus : Un site Web géré par le gouvernement fédéral
- Manuel Merk sur les saignements utérins anormaux associés à des troubles ovulatoires (AUB-O)
